# Interventions addressing impacts of climate change on sexual and reproductive health and rights in sub-Saharan Africa: A scoping review

**DOI:** 10.1371/journal.pone.0329201

**Published:** 2025-08-11

**Authors:** Jacinter A. Amadi, George Odwe, Francis Obare, Betsy Sambai, Beth Kangwana

**Affiliations:** 1 Population Council, Nairobi, Kenya; 2 Department of Plant Sciences, Kenyatta University, Nairobi, Kenya; Institute of Tropical Medicine / University of Antwerp, BELGIUM

## Abstract

Sub-Saharan Africa is faced with triple challenges of high vulnerability to climate change impacts, high levels of inequality, and poor sexual and reproductive health and rights (SRHR) outcomes. Climate change impacts can worsen the SRHR situation for high-risk groups such as women, children, adolescent girls, and people living with Human Immunodeficiency Virus (HIV). This scoping review examined interventions addressing the impacts of climate change on SRHR in the region to identify barriers to and facilitators of effective integration. The review followed Arksey and O’Malley’s framework for scoping reviews. Data search was conducted in peer-reviewed journal databases and from grey literature on the official websites of selected organizations. Data charting was conducted using the Population, Intervention, Comparator, Outcome tool in Covidence. There is limited evidence on interventions at the intersection of climate change and SRHR, with seven (7) documents included in the review. Maternal and Child Health, HIV prevention, and a combination of maternal and child health and family planning were the SRHR components addressed. Other components like Gender-based violence, harmful practices, and abortion care do not have targeted interventions. A siloed approach to SRHR and climate change programming impedes intervention integration. Documented interventions are implicit about climate risks, focus on impact pathways, and do not directly target SRHR. There are no interventions targeting vulnerable and marginalized groups. Limited policy integration, financial constraints, and poor SRHR recognition deter intervention integration. Effective and equitable integration requires that population growth impacts and SRHR issues be recognized and deliberate investments (research, policies, programs, interventions, and financing) put in place to address critical SRHR gaps and climate vulnerabilities to enhance resilience.

## Introduction

Access to Sexual and Reproductive Health and Rights (SRHR) is an integral component of Universal Health Coverage (UHC) and is crucial in the attainment of Sustainable Development Goals (SDGs). However, there are gaps regarding universal access to SRHR in sub-Saharan Africa (SSA) [1], and if not addressed, the SDG target 3.7 will remain a mirage. The leading SRHR gaps in the region include inadequate antenatal care resulting in increased maternal and child mortality, high Human Immunodeficiency Virus (HIV) burden, gender-based violence (GBV) prevalence, and lowest modern contraceptive use [1]. The region has maternal mortality rate estimated at 454 against the global target of 70 [2], accounts for 57% of global under-5 deaths [3] and 65% of the people living with HIV [4]. SSA is among the regions with the lowest proportion (56%) of modern contraceptive use [5] and has a 33% prevalence rates of lifetime intimate partner violence (IPV) among partnered women aged 15–49 years [6]. These SRHR challenges have far-reaching implications that affect the quality of life. Although many countries are committed to investing in UHC, advancing this target, including access to SRHR during post COVID-19 era has been challenging, especially Lower and Middle Income Countries (LMICs) [7] where progress has been hindered by scarce resources, lack of political goodwill and widespread gender inequality and rights issues [8].

Over the last decade, unprecedented climate change (CC) impacts have been witnessed across societies globally, with communities living in SSA being among the most vulnerable to CC risks [9]. Climate change impacts such as extreme heat, decreasing rainfall, drought, floods, and cyclones ravage communities across the region with negative consequences for water, sanitation and hygiene (WASH), food and nutrition, livelihood resources, and health [10]. Health-related impacts include injuries and mortalities related to climate extremes (CE), heat-related illnesses, respiratory infections, water-borne, food-borne and vector-borne diseases, malnutrition, and deteriorating mental and psychosocial health [9,11]. Inaccessible health facilities, disrupted health services, and effects on healthcare workers are predominant CC impacts on healthcare system. Alterations in temperature and rainfall patterns change seasonal prevalence of climate-sensitive vector-borne diseases such as Malaria, with an estimated US$ 4.1billion in annual cost for control and eradication [12]. Moreover, malnutrition driven by climate-related food insecurity is a major public health challenge [13]. Vulnerabilities to climate-related risks vary along gender, age, religion, socioeconomic, and disability status [14], with women, girls, and children being at the greatest risk. Research documents the negative consequences of CC on SRHR. For example, heat stress has been associated with adverse birth outcomes in Burkina Faso, CE has been linked to disrupted access to family planning or HIV care in Zambia and South Africa, and increased GBV including intimate partner violence in Kenya, while drought has been associated with increased HIV transmission rates and early sexual debut among adolescents in Lesotho [15,16].

Parties to the United Nations Framework Convention on Climate Change (UNFCCC) committed to preparing and adopting National Adaptation Plans (NAPs) that mainstream climate change into all government sectors to boost adaptive capacities, [17] in addition to, implementing ambitious economic and social transformations to limit global warming to below 2°C above the pre-industrial levels [18]. Correspondingly, the World Health Organization (WHO) developed a technical guide for effectively including health in the NAPs, which is referred to as a Health National Adaptation Plan (HNAP) to enhance health system resilience [19]. Resilience building requires that climate actions (adaptation and mitigation) incorporate the full breadth of health risks and vulnerabilities and design measures that are conscious of the wider health needs [20]. Equally, health policies and programs should incorporate climate actions, including efforts toward achieving net zero in the health sector [20].

Climate actions have health co-benefits and can improve SRHR outcomes of affected populations [21]. On the other hand, SRHR interventions can reduce vulnerabilities to CC impacts and increase adaptive capacities of individuals and communities. Implementing climate actions improves health determinants such as nutrition and diet, reduced pollution, and reduced ambient temperature, factors that positively impact physical and mental health, thus boosting SRHR outcomes such as maternal and child health outcomes, and reducing GBV and trauma [22]. Likewise, increased access to and uptake of modern contraceptives and Antenatal Care (ANC) services and reduced GBV and sexually transmitted diseases like HIV increase individual adaptive capacity and improve resilience to climate change impacts. These result when more time and resources are available for women and girls to engage in socio-economic enablers like education, income generation, employment, and access to resources and decision-making powers [15]. Despite the synergistic effect of their integration, climate change actions and SRHR interventions have historically been implemented in siloes [23]. This can reverse the benefits of integrated approaches and worsen the SRHR situation in the region facing current and future climate crises.

To date, there is limited research on the integration of CC and SRHR [16]. Even more concerning is the lack of emphasis on the application of the intersectional lens when it comes to the analysis of SRHR domains and climate risks [16,24]. Consequently, there is limited evidence to inform deliberate investments in integrated interventions addressing the impacts of CC on SRHR, especially those that relate to policies, programs, and financing at macro and microsystem levels. The rationale for this review stems from disproportionate vulnerability and often overlooked interlinkages between CC impacts and poor health and human rights issues in SSA. Thus, there is a need to address SRHR gaps and to empower women and girls affected by CC to make informed decisions and choices about their reproductive and sexual health. This scoping review aims to synthesize literature on interventions addressing the impacts of climate change on SRHR in SSA to identify barriers and facilitators to comprehensive integration of climate actions and SRHR needs in the region. The scoping review is guided by two research questions: (i) are there existing interventions addressing the impact of climate change on SRHR in the Sub-Saharan Africa? (ii) what are the barriers to and facilitators of SRHR integration in climate actions in the SSA region?

## Materials and methods

### Database search

This review aimed at identifying publications on interventions addressing the impacts of CC on SRHR in the SSA region using Arksey and O’Malley framework for scoping reviews [25]. Electronic databases were searched, and the resulting peer-reviewed journal articles and gray literature were subjected to further scrutiny. For peer-reviewed articles, a database search including PubMed, Springerlink, JSTOR, Taylor and Francis, and ScienceDirect was conducted in September 2024. A 20-year search period beginning January 2004 to August 2024 was used. A grey literature search on official websites of organizations such as UNFCCC, Centers for Disease Control and Prevention (CDC), United Nations Population Fund (UNFPA), Women Deliver, WHO, World Bank, United Nations Children’s Fund (UNICEF), World Vision International, Population Council Inc., Consultative Group on International Agricultural Research (CGIAR), United Nations Entity for Gender Equality and the Empowerment of Women (UN Women), United Nations High Commissioner for Refugees (UNHCR), International Rescue Committee and International Committee of the Red Cross was also conducted using key words (S1 Table) in addition to handsearching references of included articles. This study did not require ethical approval since human subjects were not involved.

### Search strategy and selection criteria

The search strategy employed keywords and concepts relating to CC and SRHR following definitions provided by the Intergovernmental Panel on Climate Change [26] and Starr et al., [8]. S1 Table contains the search strategy applied during document search. Documents retrieved from all the searches were organized in Zotero Software (Version 6.0.36, George Mason University, VA, USA) before uploading in Covidence (SaaS Enterprise, Melbourne, Australia) for further analysis. Following predetermined inclusion and exclusion criteria, two independent reviewers (JAA and BS) screened documents by titles and abstracts, conducted full text screening and data charting of included documents. Preferred Reporting Items for Systematic Review and Meta-Analysis extension for scoping reviews (PRISMA-ScR) checklist was used to guide the scoping review process [27].

### Inclusion and exclusion criteria

During study selection, documents reporting qualitative, quantitative, mixed method designs, opinions, commentaries, and reviews were examined. Documents were considered for full-text review if (i) they focused on climate change (including CE) and investigated at least one aspect of SRHR; (ii) they explored one or more SRHR elements and mentioned CC or CE (iii) if research or program was implemented in SSA. Studies published in English and on women, children, adolescents, youth, and other marginalized groups were included in the review. Documents were excluded if they either solely investigated or implemented climate change or SRHR interventions if they were conducted in regions other than the SSA, and if they were published in non-English languages. Abstracts or publications without full texts were also excluded.

### Data charting and synthesis

Data charting was performed following Population, Intervention, Comparator, Outcome data extraction tool in Covidence. The tool was pretested, and changes were effected before data extraction. Quality assessment was performed using a tool modified from Matanda et al., 2023 [28] (S2 Table). Data extracted comprised of document details, setting, methods employed, climate change event(s)/hazards investigated, target population and sample size, the impact of climate events, intervention(s) implemented or tested, and SRHR component(s) and outcome(s). Guided by research questions and data extraction tool in Covidence, data extraction was conducted by two reviewers and thematic exploration of SRHR components and CC risks was used during data synthesis.

## Results

**Fig 1:**
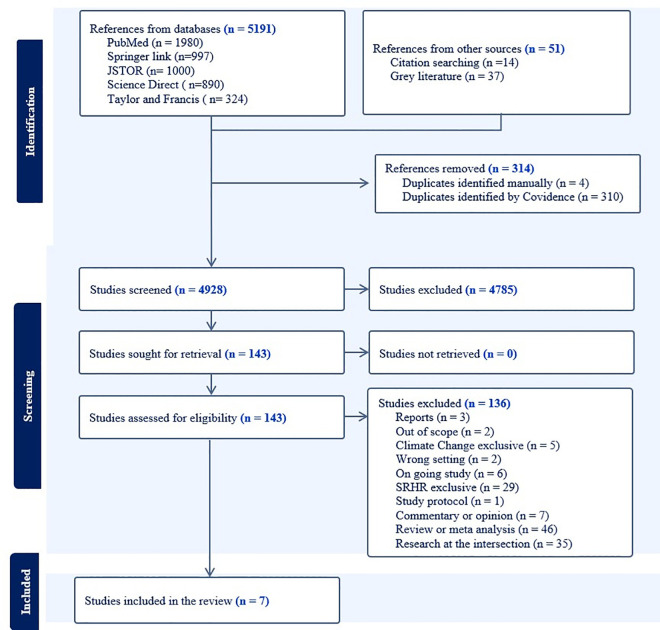
PRISMA 2020 diagram illustrating document selection, screening, and suitability assessment process.

The search generated 5,242 records from which 314 duplicates were removed. Four thousand nine hundred and twenty-eight (4,928) documents were screened by title and abstract followed by full-text evaluation of 143 documents for eligibility. Seven (7) articles including one grey lierature were retained for data extraction. Rejected documents included reviews (34%, n = 46) [29–33], research studies (26%, n = 35) [34–38], SRHR exclusive interventions (21%, n = 29) [39–42], commentaries and opinions (5%, n = 7) [23,24,43], ongoing work (4%, n = 6) [44,45], and CC exclusive interventions (4%, n = 5) [46–48]. PRISMA-2020 flowchart (Fig 1) was used to illustrate the document review process.

### Document characteristics

S3 Table summarizes the characteristics of seven (7) documents included in the review. Four (4) documents applied quantitative methods, while one (1) and two (n = 2) applied qualitative and mixed methods approaches respectively. The documents with quantitative methods used randomized control trials [49,50] and pre-post with control design [51,52]. Table 1 presents a summary of the documents included in the review. The majority of the interventions were implemented in the East Africa region, Kenya leading with four studies [49,50,52,53] followed by Tanzania [51] and Kenya and Uganda [54]. In West Africa, an article reporting on interventions conducted in Ghana was included [55].

**Table 1:** Characteristics of documents with interventions addressing impacts of CC on SRHR by type, SRHR component, and CC hazards.

Document characteristics			
Quantitative studies	57% (n = 4)		
Qualitative studies	14% (n = 1)		
Mixed methods	29% (n = 2)		
**MCH**	43% (n = 3)		
**HIV**	43% (n = 3)		
**Multiple elements of SRHR (FP and MCH)**	14% (n = 1)		
**Country**	**Rainfall changes**	**Rainfall changes and Drought**	**Multiple climate hazards**
**Kenya**	MCH [52]	HIV [49,50,53]	
**Kenya and Uganda**		FP and MCH [54]	
**Tanzania**		MCH [51]	
**Ghana**			Multiple elements [55]

Four (4) documents were of good quality, whereas two (2) were of moderate quality and one (1) was of low quality (S2 Table). The SRHR components investigated were maternal and child health (MCH) [51,52,54], HIV [49,50,53], and multiple elements [55]. An intervention program implemented in the Lake Victoria Basin of Kenya and Uganda addressed both family planning (FP) and MCH [54]. No interventions were focused on GBV or violence against women and girls (VAWG), harmful practices such as Female Genital Mutilation (FGM) and child marriage, abortion care, infertility, and reproductive cancers.

All reports documented interventions focusing on impact pathways between CC and SRHR such as agriculture and food security [49–52], food security and livelihoods [53,55], and livelihoods and income [54]. Interventions implemented included agricultural, environmental, and health, a combination of agricultural and financial, and a socio-economic intervention using cash transfers (CT). Of the seven studies, only one [55] referred to climate hazards such as rainfall, drought, floods, extreme heat, and fires. Other documents were implicit about climate change, yet the interventions employed addressed impacts related to drought, dwindling rainfall, and floods. Six documents [49–54] reported having received funding from international non-governmental organizations with only one study [55] reporting using the researcher’s source of funding.

### Interventions implemented and SRHR outcomes

Agriculture and food security intervention incorporating financial support in the form of loans for purchasing farm equipment and inputs like water pumps, fertilizers, and pesticides combined with training on sustainable agriculture and financial skills was implemented among HIV- infected adults aged 18–49 years in Kenya [49]. The intervention resulted in significant improvements in food security (p < 0.001) [49] in the intervention group. Reduction in food insecurity was in the form of increased frequency of food consumption (p = 0.013), increased body mass index (BMI) levels, and HIV virological suppression from 51% to 79% for the intervention group contrary to the control group whose virological suppression decreased [49]. Immunological analysis of absolute lymphocyte cell count showed a significant difference of 164.9 cells/mm^3^ between intervention and control participants at the end of the study [49]. Significant improvements in HIV immunological parameters were observed a year following intervention implementation. Another study conducted in rural and peri-urban Kenya reported income increases and enhanced farm productivity that resulted in improvements in HIV causal pathways including food security, mental health, physical health, and self-confidence [53]. A study comparing the effects of multisectoral agricultural interventions on HIV outcomes between widowed and married women found a significant decline (p < 0.01) in food insecurity, depression symptoms, and stigma among widows and married women in the intervention group [50]. Additionally, improvements in HIV clinic indicators (e.g., adherence to antiretroviral treatment, BMI, and viral suppression) and social support were observed within the group receiving agricultural intervention compared to the control group [50].

Interventions incorporating agricultural technologies such as crop diversification, conservation agriculture, improved animal husbandry, and land use management practices enhanced drought resilience, consequently improving nutrition among young children from participating households in Tanzania [51]. The study reported significant positive effects of agricultural technologies on the nutritional status of young children with an 18% reduction in stunting [51]. In Kenya, a study evaluating the cost of integrating health and agricultural interventions at community and facility levels reported that significant resources were needed to recruit and retain pregnant women and young children in the studies [52]. For example, to reach 3,281 women, up to 508, 000 USD was required for a 3-years project [52]. Activities such as planning, coordination, recruitment, and training across sectors escalated program costs [52].

Cash transfer components of social protection given to vulnerable households were used to manage risks related to CC like low rainfall, drought, or floods that caused livelihood loss and crop failure [55]. Families receiving conditional and unconditional CT used parts of the transfer money to do farming by purchasing farm inputs like early maturing crops, fertilizers, and pesticides that minimized losses from drought or depressed rains [55]. Some households used their CT to purchase food, livestock, or poultry especially when there was crop failure due to climate-induced drought. Some of the money was spent on needs such as health care, repair of homes, and restoration of assets destroyed by windstorms or floods [55]. Such CT interventions resulted in indirect positive SRHR outcomes such as improved health of at-risk groups such as pregnant and lactating women and children under 5 years and increased access to quality health care among households enrolled in health insurance services.

An integrated health and environmental program implemented in the Lake Victoria Basin of Kenya and Uganda incorporated multiple interventions such as outreach and training, SRHR messaging, increased contraceptive supplies, health information system strengthening, promotion of sustainable agriculture, and livelihood improvement activities [54]. These interventions resulted in enhanced adaptive capacities of local communities and improved health and well-being of women and young children in the two countries. Sexual and reproductive health (SRH) messaging and increased contraceptive supplies accelerated demand for and uptake of essential SRH services including long-acting and reversible contraceptives and facility deliveries [54]. An increase in facility delivery was observed among adolescent and young mothers aged 12–24 years [54]. Additionally, SRHR messaging increased male engagement enhancing community trust and reducing misconceptions about SRH services like FP and HIV testing. Facility-level interventions increased the accessibility of SRH services by reducing contraceptive stock-outs and increasing health worker skills notably skilled birth deliveries [54]. Institutional-level interventions resulted in better engagement with policymakers and boosted institutionalization and adoption of interventions through budget allocations and funding [54]. Training on sustainable farming practices increased food production and was a source of income for intervention groups.

## Discussion

This review documents implementation studies and interventions at the intersection of CC and SRHR, identifies barriers and facilitators of comprehensive integration, and highlights CC-SRHR components that need to be addressed to increase climate resilience and well-being. Interventions addressing CC and SRHR were implicit and neither directly referred to CC hazards nor demonstrated cause-effect relationships. This review found that most interventions addressed pathways between CC and MCH and CC and HIV with only one study reporting on a combination of interventions for FP, MCH, and environmental conservation. Interventions that explicitly mentioned the impacts of climate hazards such as extreme temperature, drought or floods on SRHR components were missing. Moreover, inventions focusing on other SRHR components such as GBV, harmful practices, fertility, and abortion care were largely absent in the literature or may have been poorly documented. A review assessing whether GBV interventions considered impacts of CC in Africa [29] reported that of the 40 GBV interventions assessed, none mentioned climate, weather, or climate change hazards and suggested that comprehensive strategies integrating GBV and CC be put in place to benefit from cost-effectiveness and synergistic impacts [29]. Gender-based violence is a product of socioeconomic and cultural vulnerabilities that are often triggered by climate-related disasters, thus there is a need for future research and interventions to focus on the occurrence, patterns, and prevention of such climate-induced violence [29].

This review finds that most interventions are still siloed. This could, in part, be due to existing gaps in addressing health in climate change policies and vice versa or inadequate documentation of program activities [23]. On a positive note, there has been an increase in the number of ongoing programs and implementation studies integrating CC and SRHR. Examples include ongoing projects in Tanzania [45] and in Madagascar, Mozambique, and South Sudan [56]. This calls for increasing approaches to health and SRHR integration into national and regional climate actions, strategies and disaster risk reduction framework and plans. Documented interventions addressed impact pathways between SRHR and CC including agriculture, food security, livelihoods, and income. Most CC adaptations focus on socioeconomic, cultural, and geographic vulnerabilities since CC impacts on SRHR are mostly indirect and the most vulnerable groups suffer additional inequalities that magnify negative SRHR outcomes. Climate-related impacts widen SRHR gaps, especially in the African region, which is yet to achieve universal access to SRHR [1]. Inaccessible ANC, post-natal care, and FP services caused by CC resulted in poor health outcomes for women and children and in unintended pregnancies and unsafe abortion [57,58] whereas disrupted HIV care and services increased HIV infections and decreased HIV suppression among persons living with HIV (PLHIV) [58]. Forced displacement and unplanned migrations increase GBV risks among women and girls, reduce bodily autonomy, and can result in poor menstrual health [59].

Integrated approaches should merge interventions addressing adverse SRHR outcomes with climate change adaptation and mitigation approaches that tackle causal pathways such as food insecurity, WASH, environmental degradation, livelihood, and income losses. This review found that interventions addressing food and nutrition insecurity have positive outcomes for child nutrition, pregnant and lactating women’s health, and HIV suppression. A combination of adaptation actions that focused on environmental conservation, sustainable agriculture, and livelihood improvements with SHRH messaging improved SRHR outcomes such as uptake and demand for FP commodities, facility deliveries, and community trust in SRHR services while at the same time improved livelihoods, income and supported climate change mitigation [54]. Such multipronged approaches should be promoted and adopted to enhance resource efficiency and gender responsive and rights-based climate actions in the region.

Interventions for social protection such as CT were integral components of SRHR programs [60,61] and CC adaptation [55]. In West Africa, CT was spent on several CC coping strategies against poor seasonal rains, drought, floods, and fires [55]. Such coping mechanisms included crop diversification and purchase of farm inputs, food and nutrition, livelihood improvements, and income diversifications. These measures had indirect positive impacts on SRHR such as improved health and nutrition of young children, pregnant and lactating women, and positive health outcomes for PLHIV. Cash transfers have great potential to support communities adapt to climate change in the medium term, especially in the SSA region that currently experiences climate extremes. The 300 billion United Nations Conference of Parties’ (COP 29) New Collective Quantifiable Goal (NCQG) on climate finance to developing countries points to the need to fully adopt CTs to support adaptation actions and to respond to loss and damage occasioned by climate extremes. Integrated CC-SRHR interventions can leverage financial mechanisms like NCQG and other global climate funding including Green Climate Fund and Global Environment Facility with potential for health co-benefits.

### Barriers to and facilitators of comprehensive integration of CC actions and SRHR needs

This review finds a siloed approach to SRHR intervention implementations and CC actions. The review also finds that while implementation research at the intersection is still in its infancy, programs addressing SRHR outcomes are on the increase [44,45]. However, some program activities are not well packaged or publicly unavailable to support scale-up and adoption [23]. In addition, analytical frameworks for examining barriers to the integration of health in climate actions and vice versa are also lacking [23]. Monitoring and evaluation of programs at the intersection is weakened by the lack of tools that capture the breadth and complexity of activities and relationships in integrated programs [54]. Consequently, there are inadequate interdisciplinary, cross-sector implementation research and weaker collaborations across disciplines.

Majority of documented interventions were donor funded. This weakens the sustainability of interventions beyond research or program implementation periods [49,53–55]. Capacity building, technology transfer and domestic and international financing coupled with effective engagement of local communities are the basis for sustainable and impactful integrated interventions [13]. Interinstitutional collaborations between local researchers and those from high-income countries, South-to-South partnerships can improve the acceptability and transferability of implementation research findings [16]. Restricted donor operations also limit possibilities of cross-sector collaboration, especially among those not considered a donor priority. Additionally, lack of policy integration [52] and recognition of SRHR needs in the government’s fiscal planning result in financial and budget constraints that limit SRHR integration as needs such as food and water security and poverty alleviation take precedence. Further, there is a dearth of information on the cost of implementing interventions for sustainability [62]. Implementation costs vary between programs and locations [62,63] and are elevated in multisectoral arrangements [55]. This can be ameliorated by leveraging on existing capacity, established systems, and infrastructure such as community health promoters [63] and extension services while employing bottom-up approaches [64].

Effective integration of health needs in climate actions will require policy adjustments in climate actions in the same breath as gender and justice integration in current CC policies [65,66]. This change will need multipronged approaches that integrate multidisciplinary tools and methodologies [67] to address interlinked challenges such as poverty, CC, and health. Such interventions should use multisectoral approaches rather than single-sector approaches. Incorporating anticipatory CT components in climate actions has the potential to support communities recover from loss and damages occasioned by extreme weather and in building adaptive capacities [68,69]. Social CT can be tailored to support climate adaptation including technology support [68] such as those used in climate-smart agriculture with co-benefits for SRHR needs like maternal and child nutrition and health. Poverty is a major hindrance to healthcare access and a cause of poor health outcomes especially among highly vulnerable groups such as women and children from rural communities and urban poor [70]. Thus, integrated interventions that address the food security-environment-health nexus can deliver a greater impact on poverty reduction by increasing food security, promoting environmental conservation, and resilience building against CC impacts.

### CC-SRHR intersections with limited targeted interventions

This review finds that most interventions address CC-SRHR impact pathways without clear linkage with CC risks. For instance, there were no interventions that directly addressed the impacts of CC risks such as rainfall seasonality or CE on SRHR outcomes. Further, this review finds that no interventions are focusing on the impacts of climate risks such as extreme heat on key SRHR components like MNCH. Yet, research has repeatedly confirmed heat-health impacts on MNCH [71,72], livable and physical labor limits across SSA [32]. A study in Kenya documented a community co-design of interventions against heat exposure on maternal and neonatal health [73]. The study found that accessibility to water points, social behavior change campaigns, and public education were considered highly sustainable and effective adaptation interventions against heat exposure [73]. Although this review reports few studies and interventions targeting women, young children, and PLHIV, vulnerable or marginalized groups including indigenous communities, adolescents and youth, persons living with disability, communities in fragile settings, and sexual minorities remain left out. The interventions documented focus on rural agrarian communities, with urban poor remaining largely under-documented.

Impacts of CE including drought and floods on SRHR outcomes such as pregnancy loss, GBV, and harmful practices like FGM and child marriage are not only inadequately addressed in research [16] but also lack interventions to support adaptation. A recent study found that GBV interventions do not consider CC impacts [29] although linkages between climate-related disasters and intimate partner violence [74], VAWG [75], and violence against children [31] are well documented. On a positive note, CC actions are beginning to recognize the need to tackle climate-induced violence such as VAWG [76]. Likewise, future robust SRHR and GBV programming should consider integrating CC actions to support resilience building in both development and humanitarian settings [76]. Since CC increases the frequency and intensity of disasters, integrating disaster risk reduction measures and climate adaptation measures should be considered a priority to enable efficient resource use and minimize duplication of effort [67]. Such interlinkages should be multidisciplinary, intersectoral, and holistic and should employ right-based approaches [77]. The existing absence of interventions focusing on CC impacts on other SRHR components including, fertility, reproductive cancers, pregnancy loss, and abortion ought to be urgently addressed. The said efforts will accelerate the integration of CC and SRHR to support the attainment of good health and well-being for all.

### Limitations

This scoping review presents findings on the extent of SRHR-CC integration and existing implementation research gaps in SSA. The review focused on reports and interventions published in English and excluded publications in other languages. Some SRHR interventions did not explicitly refer to climate hazards or related risks thus affected documents turn out when searching using climate change related terms. The focus on impact pathways may have resulted in some program interventions being left out due to restrictive search terms used.

## Conclusions

This scoping review maps existing interventions addressing CC impacts on SRHR and presents barriers and opportunities for future integration in SSA. Interventions addressing the impacts of CC on SRHR remain inadequate affecting access to SRHR services and climate change adaptation by millions of women, children, and vulnerable communities in the region. However, there is a recent increase in recognition of the interlinkages, SRHR integration in climate change actions, and a call for SRHR interventions to incorporate climate change actions. This suggests a growing interest in CC-SRHR intersectional research and programming. The diversity of SRHR components and CC risks calls for targeted interventions to minimize adverse CC impacts on SRHR. With only 12% of the SDG currently on track, the 2025–2030 period provides a critical window to fast-track integration of SRHR in climate actions and vice versa. This will deliver twin results of limiting global warming to below 2°C and achieving universal access to SRHR.

## Supporting information

S1 Table Strategy applied during document search. (DOCX)

S2 Table Quality assessment tool and outcome (modified from Matanda *et al*., 2023). (DOCX)

S3 Table Characteristics of the documents included in the review. (DOCX)
