# Supplementary material for: Interventions addressing impacts of climate change on sexual and reproductive health and rights in sub-Saharan Africa: A scoping review
Source: PLoS One. 2025 Aug 11;20(8):e0329201. doi: 10.1371/journal.pone.0329201 (PMC12338821; doi:10.1371/journal.pone.0329201)
Supplement: S1 Table — (DOCX) [file pone.0329201.s001.docx]

**S1 Table. Strategy applied during document search**

| **Key words*** | **Search terms** | **Data base** |
| --- | --- | --- |
| Climate Change, Extreme Weather Events, Sexual and Reproductive Health and Rights, Interventions, sub-Sahara Africa | "climate change" OR "climate" AND "change" OR "climate change" OR "extreme weather" OR "extreme" AND "weather" OR "climate crisis" OR "climate" AND "crisis" AND "sexual health" OR "sexual" AND "health" OR "sexual health "OR "sexual" AND "reproductive health" OR "reproductive" AND "health" OR "reproductive health" AND rights AND "Africa" OR "Africa" AND ("2010/01/01"[PubDate]: "2024/04/30"[PubDate] | PubMed (n=1980) |
|  |  | Springer Link (n=997) |
|  |  | JSTOR (n=1000) |
|  |  | Science Direct (n=890) |
|  |  | Taylor and Francis (n=324) |

Note: *Key words were applied during grey literature search.
