# Supplementary material for: Interventions addressing impacts of climate change on sexual and reproductive health and rights in sub-Saharan Africa: A scoping review
Source: PLoS One. 2025 Aug 11;20(8):e0329201. doi: 10.1371/journal.pone.0329201 (PMC12338821; doi:10.1371/journal.pone.0329201)
Supplement: S2 Table — (DOCX) [file pone.0329201.s002.docx]

**S2 Table. Quality assessment tool and outcome (modified from Matanda *et al*., 2023)**

| **Category** | **Evidence type** | **Documents appraised** | **Quality outcome** |
| --- | --- | --- | --- |
| I | A well-designed randomized control trial (RCT) | Weiser et al., 2015 [42]  Odhiambo et al., 2023 [43] | ↑ |
| II | A non- randomized study that is well-designed including a control group (e.g., quasi-experimental, matched case-control studies, pre-post with control group) | Larsen and Lilleor, 2017 [44]  Levin et al., 2019 [45] | ↑ |
| III | A study/report without randomization and does not include control group (pre-post, cohort, time series/interrupted time series, repeated cross-sectional studies) | Path International 2018 [47] | ↓ |
| IV | A well-designed non-experimental study/report including qualitative studies, and/or analysis of secondary data from reputable sources | Daniel et al., 2023 [46]  Yiridomoh et al., 2021 [48] | → |

**Key:**

↑ Good quality

→ Moderate quality

↓ Low quality
