# Supplementary material for: Interventions addressing impacts of climate change on sexual and reproductive health and rights in sub-Saharan Africa: A scoping review
Source: PLoS One. 2025 Aug 11;20(8):e0329201. doi: 10.1371/journal.pone.0329201 (PMC12338821; doi:10.1371/journal.pone.0329201)
Supplement: S3 Table — (DOCX) [file pone.0329201.s003.docx]

**S3 Table. Characteristics of the documents included in the review**

| **No.** | **Study (Setting)** | **Aim(s)** | **Climate change risk/impacts** | **SRHR component and target population** | **Design** | **Intervention(s)** | **SRHR Outcomes** |
| --- | --- | --- | --- | --- | --- | --- | --- |
| 1. | Weiser *et al*., 2015 (Kenya) [42] | To assess the effect of agricultural and finance intervention on health outcome among HIV positive adults in Kenya. | Drought and Rainfall seasonality - impacts on agriculture and food insecurity (FI) | HIV  (men and women aged 18-49 living with HIV) | RCT | Maisha Shamba intervention with three arms:  1. loan program for accessing finances  2. money maker pump for irrigation  3. Agricultural and financial training. | 1. The intervention contributed significantly to viral load suppression among intervention group (from 51% to 79%).  2. Mean CD4 cell counts increased by 75.6 cells/mm3 in intervention group while for control participants mean CD4 cell counts decreased by 89.3 at endline. |
| 2. | Pathfinder International, 2018 (Uganda and Kenya) [47] | 1. To reduce threats to biodiversity and ecosystems of Kenya and Uganda while increasing access to sexual and reproductive health services. | Drought and rainfall seasonality- impact pathway- food insecurity and livelihood | FP  MNCH  (community-wide project) | Qualitative: trainings and advocacy | PHE model that included  **1. Individual/Household (HH) level interventions:**  i) Capacity building of PHE champions and model HH to engage in and promote healthy behaviors, natural resource management (NRM) and alternative livelihoods.  ii) SRHR Messaging on healthy timing and spacing of pregnancies and MNCH  iii) Training on climate smart agriculture (CSA)  **2. Community level interventions:**  i) Training of health workers  on comprehensive FP service delivery, including LARCs, and on Emergency Obstetric and Newborn Care (EmONC) and Lifesaving Skills (LSS).  iii) Increasing contraceptive supplies and strengthening Health Information Management Systems (HMIS). | 1. Increased demand for and uptake of essential SRH services like FP and facility deliveries especially among young mothers (12-24 years).  2. Increased uptake of long-acting and reversible contraceptives (LARCs), including implants and IUDs.  3. Increased FP knowledge among boys and men.  4. Increased community trust in MNCH care that led to more pregnant women accessing MNCH services. |
| 3. | Larsen and Lilleor, 2017  (Tanzania) [44] | To assess the impact on early childhood nutrition, measured as height-for-age, of an agricultural intervention that improved food security among smallholder farmers. | Drought and rainfall indirect impact through agriculture and food security | MNCH  (children under 5 years) | Non-randomized experimental design | Rural Initiatives for Participatory Agricultural Transformation (RIPAT) intervention including crop diversification, perennial crops, conservation agriculture, improved animal husbandry, and land use management. | The RIPAT intervention improved drought resilience among the participating HHs and reduced stunting by 18% among young children. |
| 4. | Daniel et al., 2023  (Kenya) [46] | To explore the influence of access to and control over land on agricultural productivity, investments, and benefits. | Drougt and rainfall seasonality impact through food insecurity | HIV  (PLHIV aged 18-64 years) | Qualitative design | Agricultural intervention including a water pump, and farm inputs (fertilizers and pesticides); financial facilities and training on sustainable agriculture and financial literacy. | Increased income and enhanced farm productivity that resulted in improvements in HIV causal pathways including food security, mental health, physical health, and an improved self-confidence. |
| 5. | Levin et al., 2019  (Kenya) [45] | To improve the health status of pregnant women and the nutritional  status of children up to 2 years through an integrated, orange-fleshed sweet potato (OFSP) and health service delivery strategy. | Rainfall seasonality impacts through agriculture and food insecurity | MNCH  (Pregnant women attending ANC and infants < 2 years) | Non-randomized experimental design | Integrated agriculture and health intervention (Mama SASHA) focused on the promotion of  orange-fleshed sweet potato (OFSP) production and consumption and a standard APHIA training and sensitization on Infant and Young Child Nutrition services. | Improved health status of pregnant women and nutritional  status of children. |
| 6. | Yiridomoh et al., 2021  (Ghana) [48] | To minimize climate change impacts through access to social services and opportunities (social cash transfer). | Multiple: Floods, Drought, Extreme heat, Fire impacts through Lost livelihoods and income. | Most vulnerable (persons above 65 yrs, (OVC), children living with HIV, PWD, pregnant and lactating women and children under 1 year. | Cross sectional design | Livelihood Empowerment Against Poverty (LEAP) cash transfer (CT). | The CT was used to manage risks related to CC such as food and nutrition needs and health care with benefits among women and children. |
| 7. | Odhiambo et al., 2023  (Kenya) [43] | Comparison of HIV health and mental health outcomes  between widowed and married women after an agricultural intervention. | Drought and rainfall seasonality indirect impacts through food insecurity and livelihood | HIV  (married and widowed women living with HIV) | RCT | Agricultural inputs (fertilizers and pesticides).  Financial support- Loans to buy irrigation pump.  Training on agricultural and financial skills. | Significant decline in HH food insecurity, depression symptoms, and stigma.  Improvements in HIV clinic indicators for widowed and married women. |
